# Nitric Oxide Synthase Gene Transfer Overcomes the Inhibition of Wound Healing by Sulfur Mustard in a Human Keratinocyte *In Vitro* Model

**DOI:** 10.5402/2012/190429

**Published:** 2012-11-14

**Authors:** Hiroshi Ishida, Radharaman Ray, Jack Amnuaysirikul, Keiko Ishida, Prabhati Ray

**Affiliations:** ^1^Division of Experimental Therapeutics, Walter Reed Army Institute of Research, 503 Robert Grant Avenue, Silver Spring, MD 20910-7500, USA; ^2^Cellular and Molecular Biology Branch, Research Division, US Army Medical Research Institute of Chemical Defense, Aberdeen Proving Ground, MD 21010-5400, USA

## Abstract

Sulfur mustard (SM) is a chemical warfare agent that causes extensive skin injury. Previously we reported that SM exposure resulted in suppression of inducible nitric oxide synthase (iNOS) expression to inhibit the healing of scratch wounds in a cultured normal human epidermal keratinocyte (NHEK) model. Based on this finding, the present study was to use adenovirus-mediated gene transfer of iNOS to restore the nitric oxide (NO) supply depleted by exposure to SM and to evaluate the effect of NO on wound healing inhibited by SM in NHEKs. The effect of the iNOS gene transfer on iNOS protein expression and NO generation were monitored by Western blot and flow cytometry, respectively. Wound healing with or without the iNOS gene transfer after SM exposure was assessed by light and confocal microscopy. The iNOS gene transfer via adenovirus resulted in overexpression of the iNOS and an increase in NO production regardless of SM exposure in the NHEK model. The gene transfer was also effective in overcoming the inhibition of wound healing due to SM exposure leading to the promotion of wound closure. The findings in this study suggest that the iNOS gene transfer is a promising therapeutic strategy for SM-induced skin injury.

## 1. Introduction

Sulfur mustard (SM), bis-2-(chloroethyl) sulfide, is a chemical warfare agent that causes extensive skin injury. The mechanisms underlying SM-induced skin damage have remained largely unclear. The injuries may take several months to heal and they cause substantial functional and cosmetic deficits, often leading to severe disability. There are currently no standardized casualty management strategies to minimize these deficits [1]. Skin-wound healing is a complex process involving a dynamic series of events (clotting, inflammation, granulation tissue formation, epithelialization, neovascularization, collagen synthesis, and wound contraction), all associated with spatiotemporal secretion of various cytokines [2].

Keratinocytes play a fundamental role in skin metabolism and wound closure by migrating and proliferating to compensate for superficial cell loss or to cover the exposed connective tissue, and by secreting various mediators including cytokines, chemokines, and nitric oxide (NO) [3]. Frank et al. [4] reported that the enhanced induction of vascular endothelial growth factor (VEGF) expression observed in keratinocytes after cytokine stimulation was dependent on the presence of endogenously produced NO during wound healing *in vitro*. Another example is that keratinocyte growth factor (KGF), a potent mitogen for keratinocytes is enhanced by an NO donor drug, meaning that NO regulates the growth factor expression during wound healing [5]. NO also modulates the level of certain chemoattractant cytokines such as interleukins and transforming growth factor (TGF) *β*1, which initiate post-wound inflammation, resulting in promotion of keratinocyte recruitment to wounds, proliferation, and differentiation [6]. These facts strongly suggest that NO plays an important role in skin-wound healing. Recently, we reported that the level of inducible nitric oxide synthase (iNOS) peaks 24–48 h after wounding followed by completion of wound healing, but that SM exposure strongly reduces iNOS protein and mRNA expression, inhibiting wound healing in NHEKs [7]. NO is short-lived with a half-life of a few seconds. It is produced by a group of enzymes known as nitric oxide synthase (NOS). NO is a free oxygen radical and can act as a cytotoxic agent in pathological processes, particularly in inflammatory disorders [8]. These facts suggest that NO is an important signaling molecule that acts in many tissues not only to regulate a diverse range of favorable physiological cellular processes including wound healing, but it also enhances tissue damage leading to disease development [9], making it a double-edged sword. Three major NOS isoforms have been characterized, but only iNOS is stimulated by a variety of cytokines, growth factors, and inflammatory stimuli in target cells, leading to the release of much higher levels of NO (in range of *μ*mol/L). Its NO production level is 2-3 orders of magnitude greater than the levels released by constitutive NOS such as endothelial NOS or neuronal NOS [4].

The benefits of adenoviral vectors in gene transfer include relatively high transduction efficiencies, the transfection of both replicating and nonreplicating cells, and the high titers of adenovirus that can be produced [10]. The aim in the present study was to use adenovirus-mediated gene transfer of *iNOS* to restore the NO supply depleted by exposure to SM and to evaluate the effect of NO on wound healing inhibited by SM in NHEKs.

## 2. Materials and Methods

### 2.1. Materials

*Frozen* NHEKs in CryoTubes (Thermo Fisher Scientific, Waltham, MA) from single adult donor were *shipped* from Lonza (Walkersville, MD) on dry ice. Upon receipt, they were stored in a liquid nitrogen freezer. Keratinocyte growth medium (KGM, Lonza), and human keratinocyte growth supplement (KGM SingleQuots, Lonza) were also obtained from Lonza. The antibodies used in this study were as follows: (1) rabbit anti-human iNOS polyclonal antibody was obtained from Santa Cruz Biotechnology (Santa Cruz, CA); (2) mouse anti-human *β*-actin monoclonal antibody was purchased from Ambion (Austin, TX); (3) secondary antibodies (iNOS and actin) were purchased from Jackson ImmunoResearch Labs, Inc. (West Grove, PA). ECL Plus Western blotting detection reagents were obtained from GE Healthcare Bio-Sciences (Piscataway, NJ). All other reagents were purchased from Sigma (St. Louis, MO).

### 2.2. Cells and Cell Culture

Third-passaged NHEKs were used for each experiment in this study with keratinocyte basal medium containing KGM SingleQuots supplements. In brief, the original frozen cells were thawed and cultured into five 75-cm^2^ culture flasks (1st passage). When the cells became confluent, they were subcultured into fifteen 75-cm^2^ culture flasks (2nd passage). Next, the cells were collected by trypsinization and aliquoted into CryoTubes at a cell density of 2 × 10^6^ cells/mL freezing solution (Lonza)/tube. The tubes were stored in a liquid nitrogen freezer until use for each experiment. In all experiments, the 3rd passaged frozen cells were first thawed and seeded in a 75-cm^2^ culture flask. After being confluent, the cells were collected as described above, counted, and seeded onto 22 mm round collagen-coated coverslips (BD *Biosciences*, Bedford, MA) in six-well culture plates or 10-cm culture dishes at a density of 0.05 or 0.8 × 10^6^ cells per well, respectively. 

### 2.3. Construction of Adenoviral Vector Encoding Human iNOS (Ad-iNOS) and Titration of the Recombinant Adenovirus

The human iNOS gene sequence was retrieved from the NCBI GeneEntrez database. The entire coding region of the gene consisted of the Kozak sequence and both BgIII and NotI cloning sites were synthesized using the GeneOptimizerVR expert software system at Geneart AG (Germany). The synthesized cDNAs were shipped to Qbiogene (Quebec, Canada) and cloned into the vector pAdenoVator-Cmv5 (Cuo)-IRES green fluorescent protein (GFP) followed by construction of a recombinant adenovirus. The adenovirus amplification was also done with the use of 293 cells followed by purification with CsCl gradient by Qbiogene. The recombinant adenovirus that encodes the human iNOS driven by the cytomegalovirus (CMV) promoter was generated by an outsourcing company (Qbiogene). Briefly, pAdenoVator-CMV5 (CuO)-IRES-*GFP* is an adenovirus transfer vector designed for controllable gene expression used in the AdenoVator system (Qbiogene) to generate recombinant adenoviruses encoding iNOS, GFP, and the SV40 late poly(A) signal. A recombinant empty adenoviral vector containing a CMV promoter with no known gene (Ad-null: ANVP) was used as a negative control. The recombinant adenovirus preparation was titrated by Qbiogene based on optical density and 50% tissue culture infective dose (TCID_50_).

### 2.4. *In Vitro* Wounding (Scratching)

Upon reaching 100% confluence (no extra spaces between cells as observed under an inverted microscope) on either type I collagen-coated 100-mm culture dishes or 22-mm round coverslips, the medium in NHEK cultures was changed. Sixteen hours after changing medium, a wound was made by scraping an 8-channel pipette (with 200-*μ*L tips) 15 times across the 100-mm dish or twice across the round coverslip, according to a method modified from that described previously [6]. After wounding (scratching), cells were further incubated at 37°C and 5% CO_2_. Three independent experiments with duplicate dishes were carried out for data acquisition followed by analysis.

### 2.5. Sulfur Mustard Exposure with or without Adenovirus Transfection

Immediately after wounding, the medium bathing the NHEK culture was replaced with KGM containing 20 *μ*M SM as described elsewhere [7, 11] either with or without AVIP. Sulfur mustard-exposed cells remained inside a total exhaust chemical hood for 60 minutes to allow off-gassing and hydrolysis of SM to a nontoxic level followed by returning to a CO_2_ cell culture incubator. These operations were carried out at the US Army Medical Research Institute of Chemical Defense, APG, MD as described previously [7]. The medium was changed 24 h after SM exposure with fresh KGM.

### 2.6. Western Blot Analysis

Cells were washed with phosphate-buffered saline (PBS), pelleted, and lysed in the lysis buffer (M-PER Reagent; Pierce, Rockford, IL) containing proteinase inhibitors (Complete: Roche, Nutley, NJ). Cellular protein (10 *μ*g) was loaded in each lane of a 4–12% polyacrylamide-SDS gel (SDS-PAGE: NuPage, Invitrogen, Carlsbad, CA), electrophoresed at 135 volts for 90 minutes, and electrotransblotted onto polyvinylidene fluoride membranes at 35 volts for 60 minutes. Blots were incubated with the appropriate primary antibody (human iNOS rabbit polyclonal or anti-*β* actin monoclonal antibody) at a dilution of 1 : 500 to 1 : 1,000 for overnight at 4°C. Then, the membrane was subjected to the horseradish peroxidase-conjugated goat anti-rabbit or anti-mouse secondary antibodies for 30 minutes at room temperature. Proteins were visualized by the enhanced chemiluminescence (ECL) protocol (GE Healthcare Bio-Sciences). After the ECL reaction, the bands on the membrane were captured by an LAS3000 Phosphorimager (Fujifilm Medical Systems, Stamford, CT).

### 2.7. Nitric Oxide Determination

Nitric oxide was detected in the cells using diaminofluorescein-2/diacetate (DAF-2/DA) (Sigma) according to the manufacturer's protocol by fluorescence-activated cell sorting (FACS) analysis [12]. At the end of cell culture, the medium was replaced with fresh medium containing 10 mM DAF-2DA and incubated for 3 h at 37°C. Then, the cells were collected by trypsinization into a 15-mL conical tube followed by washing twice with PBS and resuspended in 1 mL PBS for FACS analysis. A BD FACS Calibur analyzer (BD Biosciences, Franklin Lakes, NJ) was used to quantify fluorescence (excitation wavelength of 488 nm and emission wavelength of 530 nm) at the single-cell level, and data were analyzed using BD FACStation Software version 6.0.2 software (BD Biosciences). Fluorescence data are expressed as mean fluorescence (percentage of control with control adjusted to 100%).

### 2.8. Wound Healing Area Measurements

In a 100% confluent NHEK monolayer culture, wounding was done using a sterile 200 *μ*L pipette tip by a vertical and a horizontal scratch intersecting each other. The intersection of vertical and horizontal scratched areas was brought to the center of the field under a microscope for all images. The pictures were taken with a digital camera (MicroFire-Model S99809, Olympus America, Center Valley, PA) under Nikon Diaphoto microscope (5x objective and 10x eye piece) (NIKON cooperation, Japan) both immediately and 24 h after scratching with or without SM treatment at the shutter speed “automatic” defaulted by Olympus. The measurements of the open areas over time with different treatments were carried out by TScratch, a software, designed specifically for the monolayer wound healing assay [10].

### 2.9. Immunofluorescence Staining of NHEK

At the end of experiment, the cells grown on coverslips were fixed with 4% paraformaldehyde in PBS and permeabilized afterwards with 0.2% (w/v) Triton X-100. Coverslips were then blocked with 1% goat normal serum in PBS followed by an overnight incubation with primary antibody (anti-iNOS) in PBS at 4°C. Next, the coverslips were washed three times with PBS and further incubated with secondary antibody (goat anti-mouse IgG-Tetramethyl Rhodamine Iso-Thiocyanate (TRITC) from Jackson ImmunoResearch Laboratories (West Grove, PA). The coverslips were then mounted onto the slides using a fluorescent mounting medium with 4′,6-diamidino-2-phenylindole (DAPI) (Vector Laboratories, Burlingame, CA). All fluorescent images were examined using a confocal microscopy system (Bio-Rad, Hercules, CA) by setting the focal plane between the bottom and top of the cells.

### 2.10. Statistical Analysis

Comparisons between the 2 experimental groups were performed using two-tail and unpaired *t-*test by GraphPad Prism 5 (GraphPad Software, La Jolla, CA). Differences in values are considered significant if *P* < 0.05.

## 3. Results

### 3.1. The Titration of the Recombinant Adenovirus Which Encodes Human iNOS Gene

The recombinant adenovirus was tittered based on optical density and 50% tissue culture infective dose (TCID_50_) as shown in Table 1. In addition, other tests were conducted for bacteria, mycoplasma, endotoxin contamination, and replication-competent adenovirus (RCA) to ensure the integrity of the recombinant adenoviral vectors. All the resulting data indicated that the adenovirus vector was of good quality for this study.

### 3.2. Gene Transfer by Ad-iNOS Infectious Viral Particles (AIVP) Results in Overexpression of iNOS

**Figure 1:**
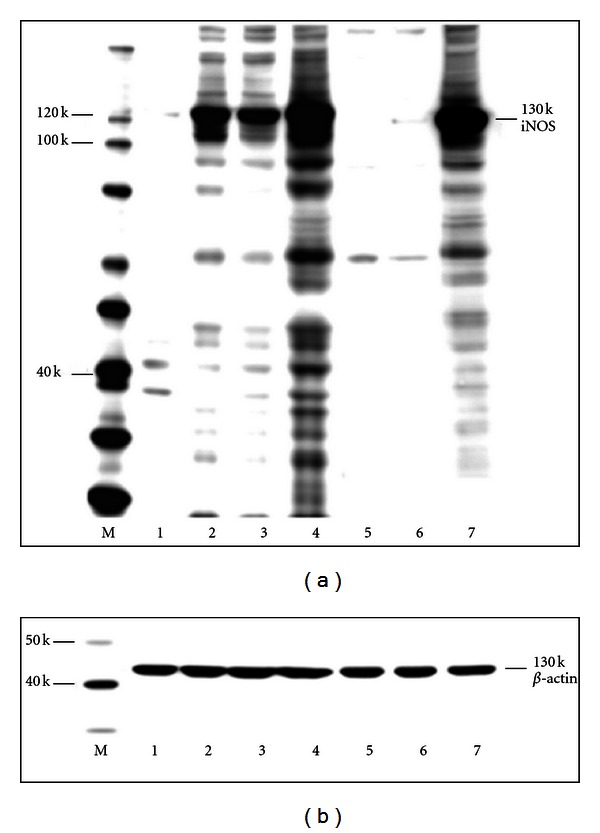
Effect of AIVP transfection on iNOS protein expression after scratching with or without SM treatment: NHEK cells were infected immediately after scratching either with AIVP or ANVP (mock) at a 1 : 5,000 dilution of the original stock shown in Table 1 immediately after scratching with or without SM treatment, and cells were then collected 24 h after scratching and subjected to Western blot analysis (a). The total cellular protein loaded in each lane was 10 *μ*g. The same PVDF membrane was reprobed with *β*-actin antibody to use as a loading control in Western blot analysis (b). The image was a representative of 3 independent Western blot experiments. Lanes: 1: control (ANVP transfection, no scratch, no transfection, no SM); 2: AIVP transfection (no scratch); 3: scratch only; 4: scratch with AIVP; 5: SM only (no scratch, ANVP); 6: scratch with SM; 7: scratch (SM, AIVP); M: molecular markers.

**Figure 2:**
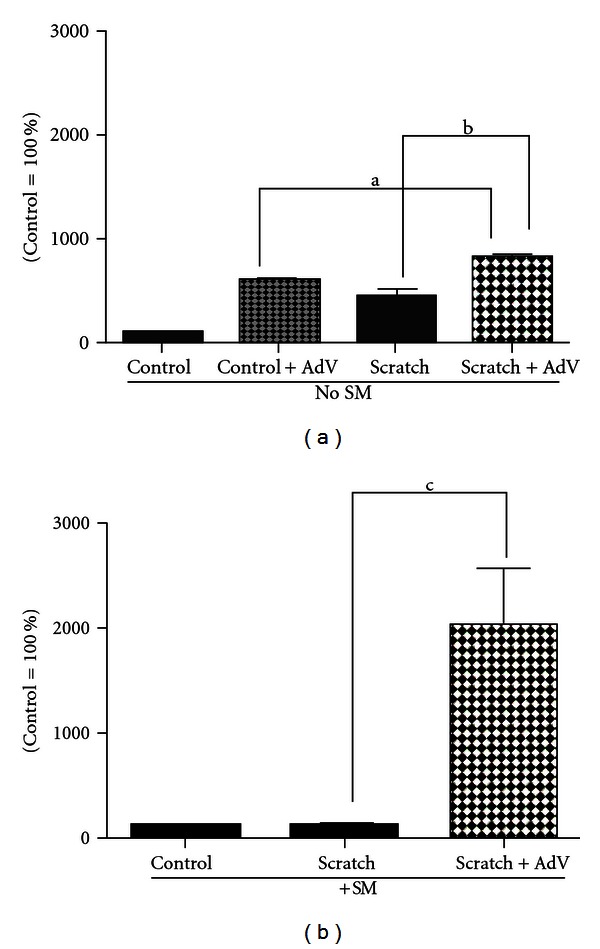
Densitometric analyses of Western blot assay for iNOS expression: For the densitometric analyses of Western blot assay (Figure 1), the iNOS expression of the control group (mock) was normalized to 100%. Values represent mean ±S.E. of 3 independent Western blot experiments. a: *P* < 0.0005; b: *P* < 0.01, and c: *P* < 0.03.

We tested the effect of the *iNOS* gene transfer by AIVP in NHEKs either without scratching or immediately after scratching on both the level of iNOS expression and NO production with or without SM treatment. The final AIVP concentration of 1 : 5,000 used for this experiment was prepared by diluting the original solution (3.83 × 10^12^ viral particles/mL, see Table 1) with KGM. Empty viral particles with no gene insertion (adenoviral-null viral particles or ANVP) were used for mock transfection with the same dilution in the control group.  Figure 1 shows a representative image of Western blot obtained on a Fuji LAS 3000 image analysis system (Fujifilm Life Science).  Figure 2 shows the summary of the densitometric analysis of the 3 independent Western blot experiments. In the control group (without both scratching and *iNOS* gene transfer), the level of iNOS protein expression showed only a trace amount as reported elsewhere. However, a strong iNOS expression was detected 24 h after scratching (Figures 1 and 2) as reported previously [7]. NHEKs without scratching but concomitant with the gene transfer expressed the equivalent amount of the protein to that of scratching only (Figures 1 and 2). The gene transfer immediately after scratching resulted in a significant additional increase of the protein expression to that of scratching only (Figures 1 and 2). However, the induction of iNOS expression by scratching only was totally abolished by SM exposure at 20 *μ*M as shown in Figures 1 and 2, which is consistent with the previous data [7]. The gene transfer of *iNOS* by AIVP immediately after scratching and SM exposure conquered the abolishment by SM with a significantly stronger expression of iNOS than that of the group with both scratching and AIVP transfection without SM treatment (Figures 1 and 2). The expression of iNOS in the control group was AIVP dose-dependent (data not shown). For each sample, 10 *μ*g of protein was loaded in SDS PAGE for the iNOS detection by Western blot. The same PVDF transfer membrane was reprobed with *β*-actin antibody for a loading control and the amount of the *β*-actin protein expressed in each sample showed no significant difference, indicating that the loaded sample in each lane was similar and thus the iNOS expression for each sample was comparable to each other (Figure 1).

### 3.3. Gene Transfer by AIVP Results in the High Output of NO in NHEKs regardless of SM Rreatment

**Figure 3:**
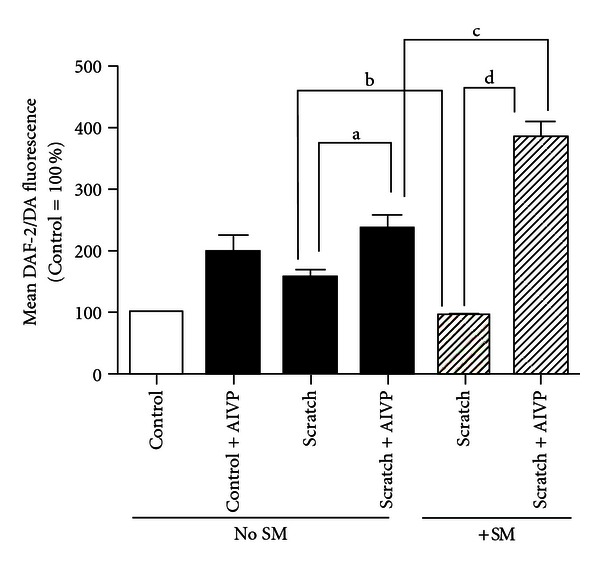
Effect of iNOS gene transfer on Nitric Oxide production measured by FACS: Nitric oxide production was measured by FACS in uninfected NHEKs and NHEKs infected with a 1 : 5,000 dilutions of AIVP 24 h after cells were scratched with or without SM treatment. Control group received ADVP transfection. The values represent mean ±S.E. of three independent experiments. a, b: *P* < 0.01; c: *P* < 0.05; and d: *P* < 0.0004.

About 2.5 times higher amount of NO production measured by FACS than that of control (no scratching) was detected 24 h after scratching without SM treatment, but this increase was abolished by SM exposure (Figure 3) as for the case of the iNOS expression (Figures 1 and 2). Without scratching, the gene transfer also resulted in the similar increased amount of NO production to that with scratching. The highest NO production was observed in the gene transfer group with scratching in spite of SM treatment as shown in Figure 3. These results were well consistent with those of Western blot analysis shown in Figures 1 and 2.

### 3.4. Light Microscopic Observation Shows That iNOS Gene Transfer Results in Resume of Wound Healing Which Is Stopped by SM Exposure

**Figure 4:**
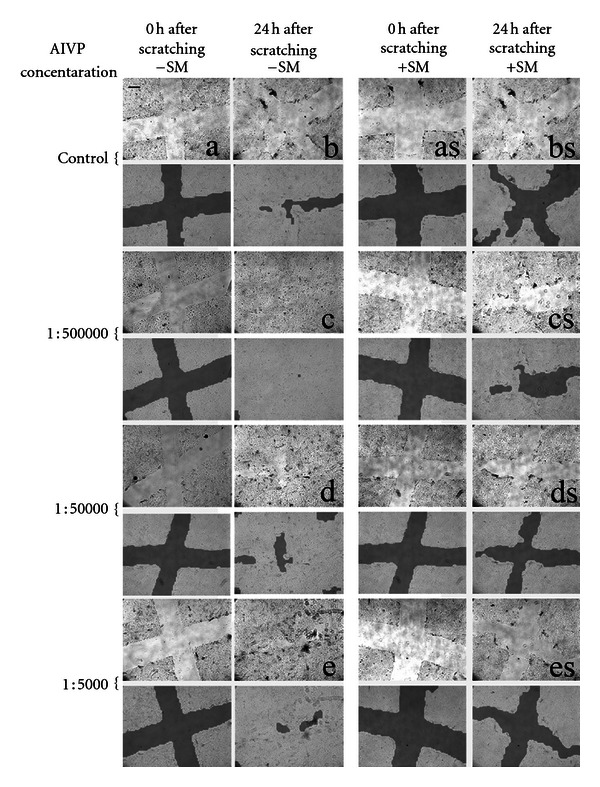
Representative light microscopic observation and scanned images by TScan with various treatments: All representative images were taken both immediately (0 h) and 24 h after scratching with or without SM treatment. Each of scanned images by TScan is shown underneath its corresponding microscopic picture. The AIVP concentrations used are displayed on the left while the treatments for each picture column are shown at the top. The scale bar on the top left panel shows 100 *μ*m. a: immediately after scratching (0 h); b: 24 h after scratching; c: 24 h after scratching with 1/500k AIVP; d: 24 h after scratching with 1/50,000 AIVP; e: 24 h after scratching with 1/5,000 AIVP; as: immediately after scratching (0 h) with SM; bs: 24 h after scratching with SM; cs: 24 h after scratching with SM + 1/500,000 AIVP; ds: 24 h after scratching with SM + 1/50,000 AIVP; es: 24 h after scratching with SM + 1/5,000 AIVP. The scale bar on the top left panel shows about 0.5 *μ*m.

**Figure 5:**
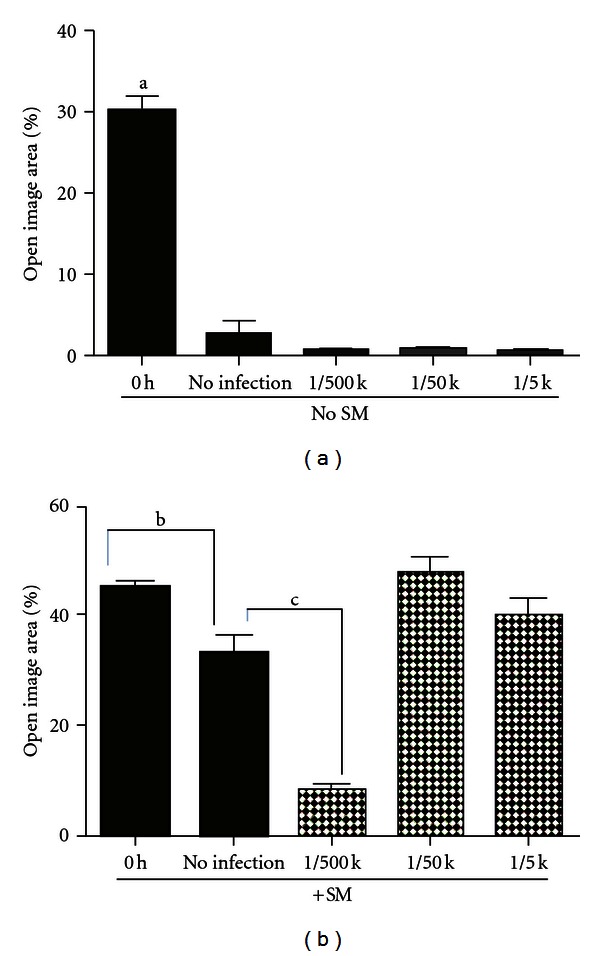
The effect of iNOS gene transfer on the rate of open area 24 h after scratching with or without SM treatment: Values indicate the mean percentage of the open area ±S.E. measured by Tscan immediately and 24 h after wounding with or without SM treatment for three independent experiments. The table shows the absolute values from three independent experiments (a and b) with duplicate cultures for each treatment. a: *P* < 0.01; b, c: *P* < 0.03.

Figure 4 shows the representative microscopic pictures of the control (ANVP), 1 : 500,000, 1 : 50,000, and 1 : 5,000 AIVP groups along with their corresponding scanned images by TScan, a software developed to access wound healing *in vitro*, for 0 and 24 h after scratching with or without SM treatment. TScan calculates the ratio (percentage) of the open space area (wounded area) against the whole image taken by a microscope. Almost complete wound healing was observed in the control (ANVP) group 24 h after scratching. Without SM treatment, the groups that received the gene transfer (1 : 500,000, 1 : 50,000, and 1 : 5,000 AIVP groups) showed the same wound healing rate as that of control (almost 100%) 24 h after scratching as shown in Figures 4 and 5 indicating no effect of the gene transfer on wound healing. On the other hand, a total inhibition of wound healing occurred with SM treatment. However, this inhibition was overcome by the gene transfer with only the lowest concentration of AIVP (1 : 500,000) tested. The higher concentration of AIVP (1 : 50,000 or 1 : 5,000) resulted in a significant increase of the rate of the open area compared to that of original wounded area, indicating its toxic effect of the gene transfer with such high concentrations of AIVP on the cells treated with SM (Figures 4 and 5).

### 3.5. Confocal Fluorescence Microscopic Observation Shows That SM Treatment Results in Inhibition of iNOS Expression after Scratching (Wounding), but the iNOS Gene Transfer Overcomes This Inhibition to Resume Wound Healing after SM Treatment

**Figure 6:**
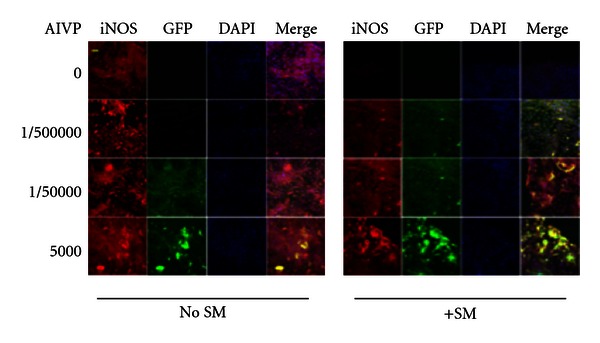
The effect of iNOS gene transfer on iNOS expression with or without SM treatment by fluorescence microscopy: The pictures are representative of five independent experiments. Red (TRITC), green (GFP), and blue (DAPI) colors represent iNOS expression, AIVP transfection, and the nucleus, respectively. The composite (merging the three colors) is shown in the fourth column for each group (±SM). The color yellow indicates that the iNOS expression was due to transfection with AIVP. The scale bar on the top left panel shows about 0.5 *μ*m.

All the images shown in Figure 6 were taken 24 h after scratching and display the effect of the gene transfer for 3 different concentrations of AIVP (1 : 500,000, 1 : 50,000, and 1 : 5,000) depicted by GFP (green) on the iNOS expression shown using TRITC (red) with or without SM treatment. Without the gene transfer and SM treatment, a strong iNOS expression (red) was observed after scratching, while no scratching resulted in no visible fluorescence signal (data not shown). Both the TRITC and GFP signals increased with the increase in AIVP concentration regardless of SM treatment. The intensity of each color (red, green, or merge) with SM treatment was stronger than that of without SM treatment (Figure 6). These data by confocal fluorescence microscopy were well consistent with those by Western blot, FACS, and light microscopy in this study.

## 4. Discussion

In this study, we hypothesized that maintaining iNOS expression by *iNOS* gene transfer after SM exposure would be a good therapeutic for skin injuries. To prove this concept, we made adenovirus constructs that encode human iNOS along with GFP as a tag. Based on the resulting data, the gene transfer was efficacious in terms of the amount of iNOS protein expression and NO production regardless of SM treatment, and was even more efficacious with SM treatment than without (Figures 1, 2, and 3).

NO is a small highly diffusible gas and a ubiquitous bioactive molecule. Its chemical properties make NO a versatile signal molecule that functions through interactions with cellular targets via either redox or additive chemistry [13]. For an example, during skin-wound healing, platelet-derived growth factor, epidermal growth, TGF-*β* and many other cytokines including VEGF are involved in restoring normal skin tissues with regard to function and appearance [10]. NO produced by NOS plays a central role in regulating these cytokines in the skin [4, 14]. Previously, we reported that NO production by iNOS is a critical factor for skin-wound healing in the same *in vitro* system used in this study. SM-treated NHEKs failed both to express iNOS after wounding and to heal, suggesting that NO production by iNOS has an important role in skin-wound healing after SM exposure [7].

About 3 times more iNOS protein expression by Western blot analysis (Figures 1 and 2), 2.5 times more NO production by FACS analysis 24 h after scratching without both scratching and SM treatment (Figure 3) were observed. These results suggested that the NHEKs were susceptible enough to AIVP used in this study. Adenovirus-mediated gene transfer has become an important tool used to introduce genetic material into cells. Yu et al. [15] reported that transduction efficiency remained greater in nonparenchymal cells than in hepatocytes after liver injury, suggesting that achieving comparable gene expression in the injured liver, higher adenoviral titers may be required. However, injured NHEKs due to SM treatment showed higher transduction efficiency with AIVP than that of intact cells. It could be interpreted that scratch triggered the signals toward generation of NO in the injured cells as presented in our previous work [7] thus the transfection of iNOS gene by AIVP resulted in “super induction” of iNOS. These findings suggest that controlled iNOS gene transferring by AIVP is a promising gene therapy for SM skin injuries. 

It should be noted that NO production has both beneficial and detrimental effects depending on the amount of NO produced by the enzyme. AIVP at the concentrations ranging from 1 : 500,000 to 1 : 5000 used in this study did not give any effect on wound healing in intact NHEKs, suggesting that AIVP were not toxic to the cells. However, for the SM exposed NHEKs, the higher concentrations at either 1 : 50,000 or 1 : 5,000 not only inhibited wound healing but worsened the healing as shown in Figure 4. On the contrary, if the lowest concentration of AIVP was used as shown in Figures 4 and 5, the gene transfer followed by enhanced NO production was beneficial to overcome the inhibitory effect of SM on the healing pathway to resume its process. The results from this study clearly showed that the higher concentrations of AIVP were detrimental to production using the lowest concentration of AIVP (1 : 5,000) was beneficial in our *in vitro* model, well explaining the general concept of NO roles in gene therapy.

The area of wound closure (healing) was measured by TScan in this study and the results from the scan seemed to be well correlated with the pictures taken by the light microscope (Figure 4). Sulfur mustard affected a molecule upstream of iNOS expression pathway such as NF-*κ*B to suppress the iNOS expression according to several studies which have reported that NF-*κ*B regulates *iNOS* expression *in vivo* and *in vitro* [16]. Their results indicate that SM induces a complex cellular response in keratinocytes, with the activation of three MAPK pathways and the NF-*κ*B pathway [17]. Gene expression profiling using a microarray analysis system, a pathway analysis, a gene ontology analysis was studied in the punch biopsies of the mouse ears exposed to SM showing complicated gene profiling after the exposure dependent on the times after exposure. The molecular mechanism of NO produced by iNOS gene transfer to promote healing in the model in this study remains to be elucidated.

### 4.1. Limitation

The application of this technology, gene transfer by virus vector in the keratinocytes, for the animal or human skin is challenging. In the future, the proof of concept for this *in vitro* study must be confirmed by *in vivo* model. *In vivo* adenoviral-mediated iNOS gene transfer is feasible, however, it would be critical and challenging to tightly control the iNOS expression within keratinocytes for the treatment of the skin injuries due to SM. Very recently, it has been nicely summarized the complex opposite outcomes of activation of iNOS in diverse contexts. As an example, iNOS promotes glioma stem cell proliferation but suppresses proliferation of T cells [18]. Those complicated actions depending upon types of cells may help explain why iNOS inhibitors or activators including NO donors have not yet been much beneficial in the clinic, and that may be the case for skin injuries with SM.

## Figures and Tables

**Table 1:** Titration of the recombinant adenovirus: The plasmid, CMV5(Cuo)-IRES-GFP (Qbiogene) containing the iNOS was used to construct and plaque purify the recombinant Ad-hiNOS-GFP adenovirus. The viral plaques were screened by Western blot analysis for expected iNOS detection followed by amplification on 3.0 × 10^9^ 293 CymR cells and purification on CsCl gradients. The recombinant adenovirus was tittered by the optical density and by TCID_50_ methods. A sterile test, detection of mycoplasma, endotoxin, and RCAs were also performed.

Test	Specification
Viral Particle (VP) Concentration	3.83 × 10^12^
Infectious Unit Concentration (TCID_50_)	1.42 × 10^11^
VP/IU Ratio	26.9
Sterile Test	Negative
Mycoplasma	Negative
Endotoxin	<0.6 U/mL
Replication-competent adenovirus (RCA)	1 RCA/1 × 10^6^ copies of viral DNA

Note: the data were provided by Qbiogene.
